# Supplementary material for: Associations Between Dietary Factors and Breast Cancer Risk: A Systematic Review of Evidence from the MENA Region
Source: Nutrients. 2025 Jan 22;17(3):394. doi: 10.3390/nu17030394 (PMC11820300; doi:10.3390/nu17030394)
Supplement: Supplementary file 1 [file nutrients-17-00394-s001.zip › nutrients-3387295-supplementary.pdf]

Table S1: Characteristics of the included studies

Reference	Country	Study designs	Study period	Sample size (case/ controls)	Study population (cases/controls)	Age (Cases/Control)	Diet assessment method	Reported exposure
Soltani et al. (2020) [1]	Iran	Case control	Between July 2013 to July 2015.	1050 (350/700)	patients with BC / healthy women (hospital-based case-control study)	65.28 ± 11.24 /61.04 ± 10.35	168-item FFQ	Dietary Approaches to Stop Hypertension (DASH)
Aghamohammadi et al. (2020) [2]	Iran	Case control	Between July 2013 and July 2015	1050(350/700)	patients with BC / healthy women (hospital-based case-control study)	65.2±11.2/ 61.04±10.3	106-item Willett-Format semi quantitative dish-based FFQ	Mediterranean Intervention for Neurodegenerative Delay (MIND) diet score
Ahmadnia et al (2016) [3]	Iran	Case control	Between 2014-2015	450(225/225)	patients with BC / healthy women (hospital-based case-control study)	36-50	40-items FFQ	Dietary patterns:<br>Bread and cereals, Dairy products, Meat and its products, Vegetables, Fruits, Miscellaneous.
Akbari et al. (2021) [4]	Iran	Case control	Between December 2019 and September 2020.	500(250/250)	patients with BC / healthy women selected from non-cancer patients of other departments (hospital-based case-control study)	47.92 ±10.33	168-items FFQ	Dietary insulin index, Dietary insulin load
Al Qadire et al. (2018) [5]	Jordan	Case control	2016	823(405/418)	patients with BC / healthy women (hospital-based case-control study)	49.2±10.2 /45.9±10.9	Self-reported questionnaire: 17 items Dietary fat screener and 7 items Fruit-vegetable screener	Dietary fat, Fruit-vegetable, Calcium supplement intake, Tomatoes intake, Garlic intake, Alcohol intake.
Alboghobeish et al. (2020) [6]	Iran	Case control	between September 2015 to February 2016	408(136/272)	patients with BC / healthy women (hospital-based case-control study)	49.4±10.6 / 47.1±10.0	168-item semi-quantitative FFQ	Carbohydrate Intake, Glycemic Index and Glycemic Load

Ali Ghalib et al. (2019) [7]	Iraq	retrospective case-control	between January 2015 and February 2019.	676(338/338)	patients with BC /women admitted in surgical ward for a wide range of elective surgical procedures (hospital-based case-control study)	47.52±11.0	Structured questionnaire	Stewed, meat, stewed chicken, Fast food, Mediterranean food, Fish, Dairy product, Yogurt, Cheese, Fruit, Vegetables, black Tea.
Alim N.E. et al. (2016) [8]	Turkey	Case control	between March and July 2016	80(40/40)	patients with BC / healthy women (hospital-based case-control study)	51.8 ± 12.90 /50.9 ± 13.05	120-items FFQ	Dietary energy, saturated fatty acid, vitamin A, vitamin E, vitamin C and fiber, Alcohol consumption.
Allahyari and al. (2022) [9]	Iran	Case control		540(180/360)			Structured questionnaire	Dietary antioxidant index (DAI)
Alothaimeen et al. (2004) [10]	Saudi Arabia	Case control	Between September 1996 and April 2002.	100(499/498)	patients with BC / healthy women (hospital-based case-control study)	44.8 ±11.5/ 36.8 ±12.8	FFQ	Triglycerides, Cholesterol, Total energy from fat, Total protein, Total fat, Polyunsaturated fat, Polyunsaturated fat, Cholesterol.
Bidgoli et al. (2014) [11]	Iran	Case control	Between 2010 to 2012	176(60/116)	premenopausal women with BC / healthy women (hospital-based case-control study)	36.45±7.02 / 34.2±5.7	Standardized questionnaire	Supplements Calcium, Supplements VitD, Dietary resources of VitD: fish and eggs,
Azzeh et al. (2022) [12]	Saudi Arabia	Case control	Between June 2014 and November 2016	432(214/218)	postmenopausal women with BC / Healthy, ethnically Arab women (hospital-based case-control study)	57 ± 7.3	FFQ.	Fruits, vegetables, meat and processed meat, poultry, dairy products and beverages, coffee and black tea, fish and seafood, olive oil, green leafy vegetables, legumes, Daily bread either white or whole wheat
Bahadoran et al. (2014) [13]	Iran	Case control	Between January and July 2010	375(100/175)	patients with BC / healthy relatives of patients or patients in other wards (hospital-based case-control study)	46.2 ± 9.3/ 45.9 ± 9.4	168-item FFQ	Total dairy intake, Low-fat dairy, High-fat dairy, Fermented dairy, non-fermented dairy

Marzbani et al. (2019) [14]	Iran	Case control	between 2013 and 2015	620(212/408)	patients with BC / healthy women (hospital-based case-control study)	41.5± 6.2/ 39.5± 7.1	11- items Questionnaire for nutrition	Dairy consumption, Fruits, Fast food (reference, soft drinks, industrially produced juices, Fats and oils, Fried foods, Sweets, Low-nutritional-value snacks.
Dashti et al. (2022) [15]	Iran	Case control	between July 2013 and July 2015.	1050(350/700)	Patients with BC / healthy women (hospital-based case-control study)	65.28±11.24 / 61.04±10.35	106-item semi-quantitative dish-based FFQ	Milk, yogurt, cheese consumption, Fat diary.
Ebrahimpour-koujan et al. (2021) [16]	Iran	Case control	Between July 2013 to July 2015.	1050(350/700)	patients with BC /healthy women (hospital-based case-control study)	61±10.3 / 65.3±11.2	106-item Willett-format semi-quantitative dish-based FFQ	Dietary Calcium
Ebrahimpour-Koujan et al. (2024) [17]	Iran	Case control	Between July 2013 and July 2015.	1050(350/700)	patients with BC / patients without BC (hospital-based case-control study)	65.3 ± 11.2/ 61 ± 10.3	168-item FFQ	Healthy eating index-2010
Ceber et al. (2005) [18]	Turkey	Case control	2003	243(123/120)	patients with BC / healthy women who have similar sociodemographic qualities with the case group (hospital-based case-control study)	49.8 ± 10.0 / 48.3 ± 7.8	27-items questionnaire on nutritional factors	Vegetable oil (margarine), Olive oil, Animal fat, Meat, beef and lamb (red meat), Poultry and fish/seafood, Vegetables, Canned food, Fruits, Cereals, Alcohol
Fararouei et al. (2018) [19]	Iran	Case control	Between November 2014 and March 2016	1010(505/505)	patients with BC / patients without BC (hospital-based case-control study)	41.78±10.65/ 42±10.62	Structured questionnaire	Red meat, fish, pickles.
Vahid et al. (2018) [20]	Iran	Case control	Between March 2015 to February 2016.	293(145/148)	patients with BC / healthy women (hospital-based case-control study)	49.8±11·31/48.5±12 .00	168-items FFQ	Dietary Inflammatory Index
Vahid et al. (2022) [21]	Iran	Case control		293(145/148)	patients with BC / patients without cancer or cancer	20-80	168-items FFQ	Dietary Antioxidant Index (DAI)

					related conditions (hospital-based case-control study)			
Farhang Djafari et al. (2023) [22]	Iran	Case control	between September 23, 2017 and June 21, 2018	300(150/150)	patients with BC / healthy women (hospital-based case-control study)	46.6 ± 10.7	147-item FFQ	Mediterranean dietary quality index (MedDQI) scores
Fereidani et al (2019) [23]	Iran	Case control	between September 2015 and February 2016.	401(134/267)	patients with BC / healthy women (hospital-based case-control study)	49.49 ± 10.68 / 47.13 ± 10.08	168- item FFQ	First nutrient pattern (Vitamins B1, B2, B3, B5, B6, B9, C, Magnesium, Iron, carbohydrate, fiber, Selenium, zinc, protein, potassium, and calcium).<br>Second nutrient pattern (Vitamins B12, A and cholesterol).<br>Third nutrient pattern (Vitamin D, EPA and DHA)
Foroozani et al. (2022) [24]	Iran	Case control	Between April 2014 and March 2017	2018(1009/1009)	women with invasive BC/ cancer-free women (hospital-based case-control study)	48.74±10.48/(IDC 47.2±9.4 and ILC 50.5±10.3)	168-items FFQ	Western dietary pattern.
Ghanbari et al. (2022) [25]	Iran	Case control		300(150/150)	women who with invasive BC/ cancer-free women		27-items food questionnaire	Food-based empirical dietary inflammatory index (FDII)
Gholamalizadeh et al (2021) [26]	Iran	Case control	Between March 2018 and February 2019	540(180/360)	patients with BC / healthy women (hospital-based case-control study)	68±18 / 65±15	168-item semi-quantitative FFQ	Total fat, Cholesterol, MUFAs , Omega-3 fatty acids, Omega-6 fatty acids (g/d)
Gholamalizadeh et al. (2022) [27]	Iran	Case control		540(180/360)	patients with BC / healthy women (hospital-based case-control study)		168-item FFQ	Dietary inflammatory index
Hammad et al. (2020) [28]	Jordan	Case control	Between October 2016 and September 2017	400(200/200)	patients with BC / healthy women (hospital-based case-control study)	20–65 years	109-items FFQ.	White Bread, Whole wheat bread, Macaroni, Bulgur, Cooked beans, Falafel, Popcorn, Peas, Corn (Total), Corn (at season), Breakfast cereals, Legumes soup (at winter), Stuffed vegetables, Carrot, Fried potato, potato
Hammad et al. (2021) [29]	Jordan	Case control	between October 2016 and	400(200/200)	patients with BC / healthy women	≥20 years	109-items FFQ.	Dietary Inflammatory Index

			September 2017.		(hospital-based case-control study)			
Hayati MSc et al. (2022) [30]	Iran	Case control	Between May 2009 and January 2018	2011(1007/1004)	premenopausal women with BC / healthy women (hospital-based case-control study)	46.6±9.32/45.96±8.56	136-item FFQ	inflammatory potential of diet
Heidari et al (2018) [31]	Iran	Case control	between September 2015 to February 2016.	401(134/267)	patients with BC / healthy women (hospital-based case-control study)	49.49 ± 10.68/ 47.13±10.08	168 item semi-quantitative FFQ	Dietary patterns: Healthy dietary pattern (high positive factor loadings for fruits, vegetables, seeds, legume, fish and seafoods, whole grains, liquid oils, olive oils and olive and lack of salt intake°.<br>Unhealthy dietary pattern (sweets, soft drinks, mayonnaise, solid oils, processed meat, fried and boiled potato, pickles and salt intake)
Heidari et al. (2020) [32]	Iran	Case control	Between September 2015 and February 2016.	401(134/267)	patients with BC /women admitted in surgical ward for a wide range of elective surgical procedures (hospital-based case-control study)	≥30 years	168 item semi-quantitative FFQ	Dietary Approaches to Stop Hypertension (DASH) (Dixon᾽s DASH diet, Mellen's DASH diet index, Fung's index, and Günter's DASH diet index)
Hosseini et al. (2021) [33]	Iran	Case control	Between September 2017 and June 2018.	300(150/150)	patients with BC /women admitted in surgical ward for a wide range of elective surgical procedures (hospital-based case-control study)	46.6±10.7/ 46.6±10.7	169 item semi-quantitative FFQ	Dietary Carbohydrate, Carbohydrate quality score CQI, low carbohydrates diet score LCDS, Total carbohydrate, Glycemic load index).
Hosseinzadeh et al. (2014) [34]	Iran	Case control	between December 2012 and September 2013.	420(140/280)	patients with BC / group-matched controls without any malignancy (hospital-based case-control study)	47.6 ± 10.7 / 46.8 ± 10.4	A structured questionnaire	Fruit and vegetables, food high in fat
Jalali et al. (2018) [35]	Iran	Case control	between September 2015 and	408(136/272)	patients with BC / healthy women	49.5±10.7/ 47.1±10.1	109-items FFQ	Dietary Inflammatory Index

			February 2016		(hospital-based case-control study)			
Jalali et al. (2022) [36]	Iran	Case control	between September 2015 and February 2016.	308(136/272)	patients with BC / healthy women (hospital-based case-control study)	49.5±10.7/47.13±10.1	168-item FFQ	Dietary Total Antioxidant Capacity (TAC) by dietary ferric reducing antioxidant potential (FRAP), TAC by oxygen radical scavenging capacity (orAC).
Jamshidinaeini MS et al. (2016) [37]	Iran	Case control	between April 2013 to May 2014	270(135/135)	premenopausal women with BC / healthy women (hospital-based case-control study)	50.4±12.56 / 50.1±11.70	168-item semi quantitative FFQ	Vitamin D
Karimi et al. (2011) [38]	Iran	Case control		274(100/174)	patients with BC / healthy women (hospital-based case-control study)	30–65	168-items FFQ	Dietary patterns:<br>Healthy dietary pattern<br>Unhealthy dietary pattern
Karimi et al. (2015) [39]	Iran	Case control	Between January and July 2010	275(100/175)	patients with BC / healthy women (hospital-based case-control study)	46.2±9 / 44.3±9.2	168-item FFQ	Dietary total antioxidant capacity
Laamiri et al. (2014) [40]	Morocco	Case control	between December 2008 and December 2010	800(400cses/400)	patients with BC / healthy women (hospital-based case-control study)	45.83±11.0 / 45.51±11.25	Food questionnaire	Red meat, Processed meat, poultry, egg, fish, fruit, vegetable, cereal, milk
Zahedi et al. (2015) [41]	Iran	Case control	Between 2013and 2014	300(150/150)	patients with BC /women admitted in surgical ward for a wide range of elective surgical procedures (hospital-based case-control study)	51.61± 10.52/51.55 ± 10.27	Structured food questionnaire	Milk, Yogurt, Oil, Meat, Poultry intake, Fast food, Fried food, Vegetables, Fruit.
Maliou et al. (2017) [42]	Algeria	Case control	between January 2015 and April 2017	379(184/195)	Patients with BC / healthy women (hospital-based case-control study)	48.4 ± 10.7 /48.4 ± 11	7- item short questionnaire of dairy foods	Dairy products, Milk, Yogurt, Fermented milk, Total cheese, Spread cheese, Fresh cheese, Soft cheese, Hard cheese

Mobarakeh et al. (2014) [43]	Iran	Case control	between April and May 2009	93(53/40)	patients with stage I to III BC/ healthy women (hospital-based case-control study)	40.02±10.01 / 39.78±11.21	Dietary habits questionnaire	High fat milk, high fat yogurt, high fat cheese, use of olive/ frying/ liquid oils for cooking, use of frying oils for frying, removing fat from meat and poultry, removing chicken skin, not use of mayonnaise as salad dressing.
Mohammad zadeh et al. (2023) [44]	Iran	Case control		408(136/272)	premenopausal women with BC / healthy women (hospital-based case-control study)		168-item FFQ	Diabetes risk reduction diet (DRRDS)
Mousavi et al. (2022) [45]	Iran	Case control	between 2013 and 2015	1050(350/700)	patients with BC / healthy women (hospital-based case-control study)	62.5	106- item Willett-format semiquantitative dish-based FFQ.	Diabetes risk reduction diet (DRRDS)
Payandeh et al. (2021) [46]	Iran	Case control		300(150/150)	patients with BC /healthy women (hospital-based case-control study)	46.6 ± 10.7 / 46.6 ± 10.7	147 food items FFQ	Plant-based diet index (PDI), plant-based diet index (hPDI), Unhealthy plant-based diet index
Rigi et al. (2021) [47]	Iran	Case control	between July 2013 and July 2015.	1050(350 /700)	patients with BC /healthy women (hospital-based case-control study)	65.2±11.2 / 61.4±10.3	106-item Willettformat semi-quantitative dish-based FFQ	Plant-based diet index (PDI), plant-based diet index (hPDI), Unhealthy plant-based diet index
Rigi et al. (2022) [48]	Iran	Case control	between July 2013 and July 2015.	1050(350/700)	patients with BC / healthy women (hospital-based case-control study)	> 30	106-items Willett-format semiquantitative dish-based FFQ	dietary glycaemic index GL, dietary glycaemic load GL
Sadeghi et al. (2023) [49]	Iran	Case control	Between July 2013 and July 2015	1050(350 /700)	patients with BC /healthy women (hospital-based case-control study)	65.2 ± 11.2 /61.0 ± 10.3	106-item Willettformat semi-quantitative dish-based FFQ	Mediterranean diet scores
Safabakhsh et al. (2020) [50]	Iran	Case control	between September 2017 and June 2018	300(150/150)	patients with BC / healthy relatives of patients or patients in other wards (hospital-	24–73	147-item FFQ	dietary total antioxidant capacity

					based case-control study)			
Safabakhsh, M et al. (2021) [51]	Iran	Case control	between September 23, 2017, and June 21, 2018.	300(150/150)	patients with BC / healthy relatives of patients or patients in other wards (hospital-based case-control study)	24–73	147-item FFQ	vegetables and fruits
Sasanfar et al. (2019) [52]	Iran	Case control	Between 2014 and 2016	1009(486/523)	patients with BC /healthy visitors, relatives and friends of non-cancer pa tients (hospital-based case-control study)	46.3 ± 10.4 / 44.2 ± 11.3	168-validated FFQ	Low-carbohydrate diet (LCD)
Sasanfar et al. (2020) [53]	Iran	Case control	Between May 2014 and April 2016.	1030(503/506)	patients with BC / healthy women with no dietary restrictions for long term. (Hospital based case-control study)	46.3±10.4 /44.2±11.3	168-items FFQ	dietary total antioxidant capacity
Sasanfar et al. (2021) [54]	Iran	Case control	between 2014 and 2016	(412 / 456)	patients with BC /healthy women (hospital-based case-control study)	46.2± 10.3/ 44.2 ±11.1	168-items FFQ	Plant-based diet index (PDI), healthy plant-based diet index (hPDI), Unhealthy plant-based diet index
Sasanfar et al . (2021) [55]	Iran	Case control	between 2014 and 2016.	956(461/495)	patients with BC /women admitted in surgical ward for a wide range of elective surgical procedures (hospital-based case-control study)	46.0±10.31/ 44.05±11.26	168-item FFQ	Dietary carbohydrate
Shafie et al. (2023) [56]	Iran	Case control	in September 2020	360(120/240)	patients with BC / healthy women (hospital-based case-control study)	58.50 (51.50–66.75) /48.00 (42.25–56.00)	168-item FFQ	Dietary fat

Sharif et al. (2020) [57]	Iran	Case control	Between July 2013 and July 2015.	1050(350/700)	patients with BC / healthy women (a population-based case-control study)	65.2 ± 11.2 / 61.0 ± 10.3	106-item Willett format semi-quantitative dish-based FFQ	Legume and Nuts
Sheikhhossein et al. (2020) [58]	Iran	Case control	between September 2017 and June 2018	300(150/150)	premenopausal women with BC / healthy women (hospital-based case-control study)	46.6 ± 10.7 / 46.6 ± 10.7	147-item semi-quantitative FFQ	MIND diet
Sheikhhossein et al. (2021) [59]	Iran	Case control	between 2017 and 2018	300(150/150)	patients with BC / healthy women selected from visitors, relatives, and friends of non-cancer patients (hospital-based case-control study)	46.6 ± 10.7 / 46.6 ± 10.7	147-item FFQ	Dietary Insulin Index and Insulin Load
Sohouli et al. (2022) [60]	Iran	Case control	Between 2019 and 2020	520(253/267)	Patients with BC / healthy women (hospital-based case-control study)	48.91±10.46 /47.13±10.08	168-items FFQ	Dietary inflammation score (DIS), empirical dietary inflammatory index (EDII)
Tajaddini et al (2015) [61]	Iran	Case control	between January 2012 and June 2013	615(306/309	patients with BC, aged 25 to 65BC / women hospitalized for non-neoplastic diseases (hospital-based case-control study)	46.4±10.2/ 41.4±9.6	168-items FFQ	White bread, Whole-wheat bread, Cake (g/day), Biscuits (g/day), Bananas (g/day), Pasta (macaroni/spaghetti), Rice, white, Legumes (g/day), Potatoes (baked/boiled), Potatoes (fried).
Tayyem RF et al. (2019) [62]	Jordan	Case control	between October 2016 and September 2017	400(200/200)	patients with BC / healthy women (hospital-based case-control study)	48.9 ± 0.6 / 47.5±0.6	109-items FFQ	Nutrient pattern:<br>High vitamin C and β-carotene nutrient intake pattern<br>High calcium, phosphorus, and vitamin D nutrient intake patter<br>High-fat nutrient intake pattern
Tiznobeyk et al. (2016) [63]	Iran	Case control	between February	150(80/70)	patients with benign BC / healthy women	42.5 ±7.8 / 43.4 ±8.3	168-items FFQ	Dietary patterns:<br>Healthy dietary pattern<br>Unhealthy dietary pattern

			2014 to April 2015.		(hospital-based case-control study)			
Toklu et al. (2018) [64]	Turkey	Case control	between December 1st, 2016, and June 1st, 2017.	130(65/65)	patients with BC / healthy women (hospital-based case-control study)	26±40.0/26±40.0	FFQ	Fruits, Legumes, Use of olive oil
Toorang et al. (2022) [65]	Iran	Case control	between 2014 and 2016	984(477/507)	patients with BC /healthy women (hospital-based case-control study)	45.9±10.3 / 43.9±11.2	168-validated FFQ	Dietary Approaches to Stop Hypertension (DASH diet)

Table S2: NIH Quality Assessment Tool for Observational Studies

Reference	***Was the research question OR objective in this paper clearly stated?***	***Was the study population clearly specified and defined?***	***the research question or objective, the study population, and the justification for the sample size.***	***Were controls selected or recruited from the same or similar population that gave rise to the cases (including the same timeframe)?***	***Were the definitions, inclusion and exclusion criteria, algorithms or processes used to identify or select cases and controls valid, reliable, and implemented consistently across all study participants?***	***Were the cases clearly defined and differentiated from controls?***	***If less than 100 percent of eligible cases and/or controls were selected for the study, were the cases and/or controls randomly selected from those eligible?***	***Was there use of concurrent controls?***	***Were the investigators able to confirm that the exposure/risk occurred prior to the development of the condition or event that defined a participant as a case?***	***Were the measures of exposure/risk clearly defined, valid, reliable, and implemented consistently (including the same time period) across all study participants?***	***Were the assessors of exposure/risk blinded to the case or control status of participants?***	***Were key potential confounding variables measured and adjusted statistically in the analyses? If matching was used, did the investigators account for matching during study analysis?***	***Overall***
Soltani et al. (2020)	*Yes*	*Yes*	*Yes*	*Yes*	*Yes*	*Yes*	*Yes*	*NA*	*Yes*	*Yes*	*NR*	*Yes*	*Good*
Aghamohammadi et al. (2020)	*Yes*	*Yes*	*Yes*	*Yes*	*Yes*	*Yes*	*Yes*	*NA*	*Yes*	*Yes*	*NR*	*Yes*	*Good*

Ahmadnia et al (2016)	*Yes*	*Yes*	*Yes*	*Yes*	*Yes*	*Yes*	*Yes*	*NA*	*Yes*	*Yes*	*NR*	*NR*	*Fair*
Akbari et al. (2021)	*Yes*	*Yes*	*Yes*	*Yes*	*Yes*	*Yes*	*Yes*	*NA*	*Yes*	*Yes*	*NR*	*Yes*	*Good*
Al Qadire et al. (2018)	*Yes*	*Yes*	*Yes*	*Yes*	*Yes*	*Yes*	*Yes*	*NA*	*Yes*	*Yes*	*NR*	*Yes*	*Good*
Alboghobeish et al. (2020)	*Yes*	*Yes*	*Yes*	*Yes*	*Yes*	*Yes*	*Yes*	*NA*	*Yes*	*Yes*	*NR*	*Yes*	*Good*
Ghalib, et al. (2019)	*Yes*	*Yes*	*Yes*	*Yes*	*Yes*	*Yes*	*Yes*	*NA*	*Yes*	*Yes*	*NR*	*Yes*	*Good*
Alim, et al. (2016)	*Yes*	*Yes*	*Yes*	*Yes*	*Yes*	*Yes*	*Yes*	*NA*	*Yes*	*Yes*	*NR*	*Yes*	*Good*
Allahyari and al. (2022)	*Yes*	*Yes*	*Yes*	*Yes*	*Yes*	*Yes*	*Yes*	*NA*	*Yes*	*Yes*	*NR*	*Yes*	*Good*
Alothaimeen et al. (2004)	*Yes*	*Yes*	*Yes*	*Yes*	*Yes*	*Yes*	*Yes*	*NA*	*Yes*	*Yes*	*NR*	*Yes*	*Good*
Bidgoli et al. (2014)	*Yes*	*Yes*	*Yes*	*Yes*	*Yes*	*Yes*	*Yes*	*NA*	*Yes*	*Yes*	*NR*	*Yes*	*Fair*
Azzeh et al. (2022)	*Yes*	*Yes*	*Yes*	*Yes*	*Yes*	*Yes*	*Yes*	*NA*	*Yes*	*Yes*	*NR*	*Yes*	*Good*
Bahadoran et al. (2014)	*Yes*	*Yes*	*Yes*	*Yes*	*Yes*	*Yes*	*Yes*	*NA*	*Yes*	*Yes*	*NR*	*Yes*	*Good*
Marzbani et al. (2019)	*Yes*	*Yes*	*Yes*	*Yes*	*No*	*Yes*	*Yes*	*NA*	*Yes*	*Yes*	*NR*	*Yes*	*Fair*
Dashti et al. (2022)	*Yes*	*Yes*	*Yes*	*Yes*	*Yes*	*Yes*	*Yes*	*NA*	*Yes*	*Yes*	*NR*	*Yes*	*Good*
Ebrahimpour-koujan et al. (2021)	*Yes*	*Yes*	*Yes*	*Yes*	*No*	*Yes*	*Yes*	*NA*	*Yes*	*Yes*	*NR*	*Yes*	*Fair*
Ebrahimpour-Koujan et al. (2024)	*Yes*	*Yes*	*Yes*	*Yes*	*Yes*	*Yes*	*Yes*	*NA*	*Yes*	*Yes*	*NR*	*Yes*	*Good*
Ceber et al. (2005)	*Yes*	*Yes*	*Yes*	*Yes*	*Yes*	*Yes*	*Yes*	*NA*	*Yes*	*Yes*	*NR*	*NR*	*Fair*
Fararouei et al. (2018)	*Yes*	*Yes*	*Yes*	*Yes*	*Yes*	*Yes*	*Yes*	*NA*	*Yes*	*Yes*	*NR*	*Yes*	*Good*
Vahid et al. (2018)	*Yes*	*Yes*	*Yes*	*Yes*	*Yes*	*Yes*	*Yes*	*NA*	*Yes*	*Yes*	*NR*	*Yes*	*Good*
Vahid et al. (2022)	*Yes*	*Yes*	*Yes*	*Yes*	*Yes*	*Yes*	*Yes*	*NA*	*Yes*	*Yes*	*NR*	*Yes*	*Good*
Farhang Djafari et al. (2023)	*Yes*	*Yes*	*Yes*	*Yes*	*Yes*	*Yes*	*Yes*	*NA*	*Yes*	*Yes*	*NR*	*Yes*	*Good*
Fereidani et al (2019)	*Yes*	*Yes*	*Yes*	*Yes*	*Yes*	*Yes*	*Yes*	*NA*	*Yes*	*Yes*	*NR*	*Yes*	*Good*

Forozani et al. (2022)	*Yes*	*Yes*	*Yes*	*Yes*	*Yes*	*Yes*	*Yes*	*NA*	*Yes*	*Yes*	*NR*	*Yes*	*Good*
Ghanbari et al. (2022)	*Yes*	*Yes*	*Yes*	*Yes*	*Yes*	*Yes*	*Yes*	*NA*	*Yes*	*Yes*	*NR*	*Yes*	*Good*
Gholamalizadeh et al (2021)	*Yes*	*Yes*	*Yes*	*Yes*	*Yes*	*Yes*	*Yes*	*NA*	*Yes*	*Yes*	*NR*	*Yes*	*Good*
Gholamalizadeh et al. (2022)	*Yes*	*Yes*	*Yes*	*Yes*	*Yes*	*Yes*	*Yes*	*NA*	*Yes*	*Yes*	*NR*	*Yes*	*Good*
Hammad et al. (2020)	*Yes*	*Yes*	*Yes*	*Yes*	*Yes*	*Yes*	*Yes*	*NA*	*Yes*	*Yes*	*NR*	*Yes*	*Good*
Hammad et al. (2021)	*Yes*	*Yes*	*Yes*	*Yes*	*Yes*	*Yes*	*Yes*	*NA*	*Yes*	*Yes*	*NR*	*Yes*	*Good*
Hayati MSc et al. (2022)	*Yes*	*Yes*	*Yes*	*Yes*	*Yes*	*Yes*	*Yes*	*NA*	*Yes*	*Yes*	*NR*	*Yes*	*Good*
Heidari et al (2018)	*Yes*	*Yes*	*Yes*	*Yes*	*Yes*	*Yes*	*Yes*	*NA*	*Yes*	*Yes*	*NR*	*Yes*	*Good*
Heidari et al. (2020)	*Yes*	*Yes*	*Yes*	*Yes*	*Yes*	*Yes*	*Yes*	*NA*	*Yes*	*Yes*	*NR*	*Yes*	*Good*
Hosseini et al. (2021)	*Yes*	*Yes*	*Yes*	*Yes*	*Yes*	*Yes*	*Yes*	*NA*	*Yes*	*Yes*	*NR*	*Yes*	*Good*
Hosseinzadeh et al. (2014)	*Yes*	*Yes*	*Yes*	*Yes*	*Yes*	*Yes*	*Yes*	*NA*	*Yes*	*Yes*	*NR*	*Yes*	*Good*
Jalali et al. (2018)	*Yes*	*Yes*	*Yes*	*Yes*	*Yes*	*Yes*	*Yes*	*NA*	*Yes*	*Yes*	*NR*	*Yes*	*Good*
Jalali et al. (2022)	*Yes*	*Yes*	*Yes*	*Yes*	*Yes*	*Yes*	*Yes*	*NA*	*Yes*	*Yes*	*NR*	*Yes*	*Good*
Jamshidinaeini MS et al. (2016)	*Yes*	*Yes*	*Yes*	*Yes*	*Yes*	*Yes*	*Yes*	*NA*	*Yes*	*Yes*	*NR*	*Yes*	*Good*
Karimi et al. (2011)	*Yes*	*Yes*	*Yes*	*Yes*	*Yes*	*Yes*	*Yes*	*NA*	*Yes*	*Yes*	*NR*	*Yes*	*Good*
Karimi et al. (2015)	*Yes*	*Yes*	*Yes*	*Yes*	*Yes*	*Yes*	*Yes*	*NA*	*Yes*	*Yes*	*NR*	*Yes*	*Good*
Laamiri et al. (2014)	*Yes*	*Yes*	*Yes*	*Yes*	*Yes*	*Yes*	*Yes*	*NA*	*Yes*	*Yes*	*NR*	*Yes*	*Good*
Zahedi et al. (2015)	*Yes*	*Yes*	*Yes*	*Yes*	*Yes*	*Yes*	*Yes*	*NA*	*Yes*	*Yes*	*NR*	*NR*	*Fair*
Maliou et al. (2017)	*Yes*	*Yes*	*Yes*	*Yes*	*Yes*	*Yes*	*Yes*	*NA*	*Yes*	*Yes*	*NR*	*Yes*	*Good*
Mobarakeh et al. (2014)	*Yes*	*Yes*	*Yes*	*Yes*	*Yes*	*Yes*	*Yes*	*NA*	*Yes*	*Yes*	*NR*	*Yes*	*Good*
Mohammadzadeh et al. (2023)	*Yes*	*Yes*	*Yes*	*Yes*	*Yes*	*Yes*	*Yes*	*NA*	*Yes*	*Yes*	*NR*	*Yes*	*Good*

Mousavi et al. (2022)	*Yes*	*Yes*	*Yes*	*Yes*	*Yes*	*Yes*	*Yes*	*NA*	*Yes*	*Yes*	*NR*	*Yes*	*Fair*
Payandeh et al. (2021)	*Yes*	*Yes*	*Yes*	*Yes*	*Yes*	*Yes*	*Yes*	*NA*	*Yes*	*Yes*	*NR*	*Yes*	*Good*
Rigi et al. (2021)	*Yes*	*Yes*	*Yes*	*Yes*	*Yes*	*Yes*	*Yes*	*NA*	*Yes*	*Yes*	*NR*	*Yes*	*Good*
Rigi et al. (2022)	*Yes*	*Yes*	*Yes*	*Yes*	*Yes*	*Yes*	*Yes*	*NA*	*Yes*	*Yes*	*NR*	*Yes*	*Good*
Sadeghi et al. (2023)	*Yes*	*Yes*	*Yes*	*Yes*	*Yes*	*Yes*	*Yes*	*NA*	*Yes*	*Yes*	*NR*	*Yes*	*Good*
Safabakhsh et al. (2020)	*Yes*	*Yes*	*Yes*	*Yes*	*Yes*	*Yes*	*Yes*	*NA*	*Yes*	*Yes*	*NR*	*Yes*	*Good*
Safabakhsh, M et al. (2021)	*Yes*	*Yes*	*Yes*	*Yes*	*Yes*	*Yes*	*Yes*	*NA*	*Yes*	*Yes*	*NR*	*Yes*	*Good*
Sasanfar et al. (2019)	*Yes*	*Yes*	*Yes*	*Yes*	*Yes*	*Yes*	*Yes*	*NA*	*Yes*	*Yes*	*NR*	*Yes*	*Good*
Sasanfar et al. (2020)	*Yes*	*Yes*	*Yes*	*Yes*	*Yes*	*Yes*	*Yes*	*NA*	*Yes*	*Yes*	*NR*	*Yes*	*Good*
Sasanfar et al. (2021)	*Yes*	*Yes*	*Yes*	*Yes*	*Yes*	*Yes*	*Yes*	*NA*	*Yes*	*Yes*	*NR*	*Yes*	*Good*
Shafie et al. (2023)	*Yes*	*Yes*	*Yes*	*Yes*	*Yes*	*Yes*	*Yes*	*NA*	*Yes*	*Yes*	*NR*	*Yes*	*Good*
Sharif et al. (2020)	*Yes*	*Yes*	*Yes*	*Yes*	*Yes*	*Yes*	*Yes*	*NA*	*Yes*	*Yes*	*NR*	*Yes*	*Fair*
Sheikhhossein et al. (2020)	*Yes*	*Yes*	*Yes*	*Yes*	*Yes*	*Yes*	*Yes*	*NA*	*Yes*	*Yes*	*NR*	*Yes*	*Good*
Sheikhhossein et al. (2021)	*Yes*	*Yes*	*Yes*	*Yes*	*Yes*	*Yes*	*Yes*	*NA*	*Yes*	*Yes*	*NR*	*Yes*	*Good*
Sohouli et al. (2022)	*Yes*	*Yes*	*Yes*	*Yes*	*Yes*	*Yes*	*Yes*	*NA*	*Yes*	*Yes*	*NR*	*Yes*	*Good*
Tajaddini et al (2015)	*Yes*	*Yes*	*Yes*	*Yes*	*Yes*	*Yes*	*Yes*	*NA*	*Yes*	*Yes*	*NR*	*Yes*	*Good*
Tayyem RF et al. (2019)	*Yes*	*Yes*	*Yes*	*Yes*	*Yes*	*Yes*	*Yes*	*NA*	*Yes*	*Yes*	*NR*	*Yes*	*Good*
Tiznobeyk et al. (2016)	*Yes*	*Yes*	*Yes*	*Yes*	*Yes*	*Yes*	*Yes*	*NA*	*Yes*	*Yes*	*NR*	*Yes*	*Good*
Toklu et al. (2018)	*Yes*	*Yes*	*Yes*	*Yes*	*Yes*	*Yes*	*Yes*	*NA*	*Yes*	*Yes*	*NR*	*Yes*	*Fair*
Toorang et al. (2022)	*Yes*	*Yes*	*Yes*	*Yes*	*Yes*	*Yes*	*Yes*	*NA*	*Yes*	*Yes*	*NR*	*Yes*	*Good*

**CD, cannot determine; NA*: Not applicable; NR*: Not reported*

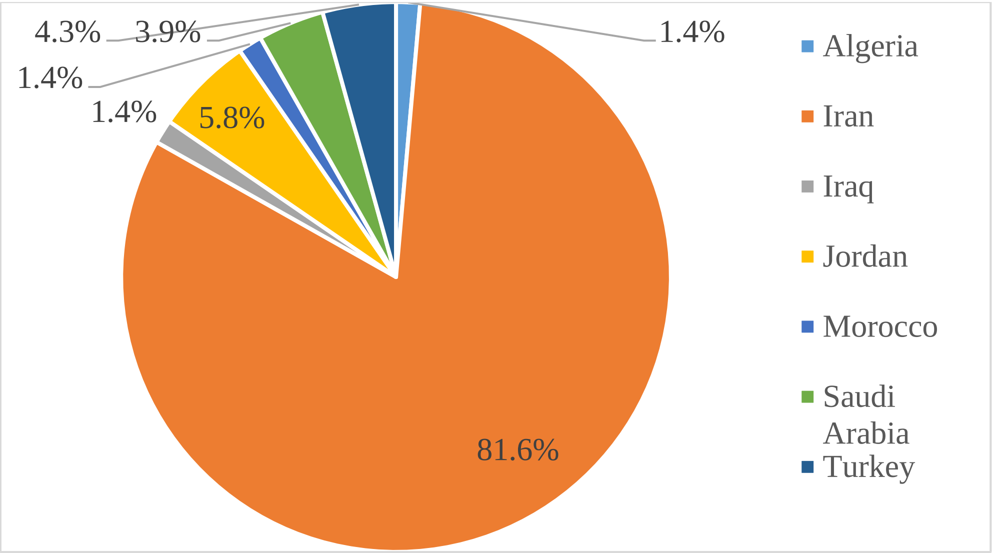

Figure S1: Geographical distribution of included studies.

Table S3: The search strategy used for sudies identification.

Data sources	Search Terms/Equations
pubmed (advenced research)	(((("breast"[MeSH Terms] OR "breast"[All Fields] OR "breasts"[All Fields] OR "breast s"[All Fields]) AND ("cancer*"[All Fields] OR "neoplasm*"[All Fields] OR "tumour*"[All Fields] OR "tumor*"[All Fields] OR "carcinoma*"[All Fields] OR "adenocarcinoma*"[All Fields])) OR (("mammaries"[All Fields] OR "mammary glands, human"[MeSH Terms] OR "human mammary glands"[All Fields] OR "mammary"[All Fields] OR "breast"[MeSH Terms] OR "breast"[All Fields]) AND ("cancer*"[All Fields] OR "neoplasm*"[All Fields] OR "tumour*"[All Fields] OR "tumor*"[All Fields] OR "carcinoma*"[All Fields] OR "adenocarcinoma*"[All Fields]))) AND ("middle east"[MeSH Terms] OR ("middle"[All Fields] AND "east"[All Fields]) OR "middle east"[All Fields] OR ("africa, northern"[MeSH Terms] OR ("africa"[All Fields] AND "northern"[All Fields]) OR "northern africa"[All Fields] OR ("north"[All Fields] AND "africa"[All Fields]) OR "north africa"[All Fields]) OR ("islam"[MeSH Terms] OR "islam"[All Fields] OR "islamic"[All Fields] OR "islam s"[All Fields] OR "islamism"[All Fields]) OR ("arabs"[MeSH Terms] OR "arabs"[All Fields]) OR ("africa"[MeSH Terms] OR "africa"[All Fields] OR "africa s"[All Fields] OR "africas"[All Fields]) OR "EMRO"[All Fields] OR ("Eastern"[All Fields] AND ("mediterranean"[All Fields] OR "mediterraneans"[All Fields])) OR "MENA"[All Fields] OR ("bahrain"[MeSH Terms] OR "bahrain"[All Fields]) OR ("egypt"[MeSH Terms] OR "egypt"[All Fields] OR "egypt s"[All Fields]) OR ("iraq"[MeSH Terms] OR "iraq"[All Fields]) OR ("iran"[MeSH Terms] OR "iran"[All Fields]) OR ("israel"[MeSH Terms] OR "israel"[All Fields] OR "israel s"[All Fields]) OR ("jordan"[MeSH Terms] OR "jordan"[All Fields]) OR ("kuwait"[MeSH Terms] OR "kuwait"[All Fields] OR "kuwait s"[All Fields]) OR ("lebanon"[MeSH Terms] OR "lebanon"[All Fields] OR "lebanon s"[All Fields]) OR ("libya"[MeSH Terms] OR "libya"[All Fields]) OR ("morocco"[MeSH Terms] OR "morocco"[All Fields]) OR ("oman"[MeSH Terms] OR "oman"[All Fields]) OR "Palestine"[All Fields] OR ("qatar"[MeSH Terms] OR "qatar"[All Fields] OR "qatar s"[All Fields]) OR ("south sudan"[MeSH Terms] OR ("south"[All Fields] AND "sudan"[All Fields]) OR "south sudan"[All Fields]) OR ("sudan"[MeSH Terms] OR "sudan"[All Fields] OR "sudans"[All Fields] OR "sudan s"[All Fields]) OR ("syria"[MeSH Terms] OR "syria"[All Fields] OR "syria s"[All Fields]) OR ("saudi arabia"[MeSH Terms] OR ("saudi"[All Fields] AND "arabia"[All Fields]) OR "saudi arabia"[All Fields]) OR ("turkey"[MeSH Terms] OR "turkey"[All Fields] OR "turkey s"[All Fields] OR "turkeys"[MeSH Terms] OR "turkeys"[All Fields]) OR ("tunisia"[MeSH Terms] OR "tunisia"[All Fields]) OR ("united arab emirates"[MeSH Terms] OR ("united"[All Fields] AND "arab"[All Fields] AND "emirates"[All Fields]) OR "united arab emirates"[All Fields]) OR ("yemen"[MeSH Terms] OR "yemen"[All Fields]))) AND ((((((((((("Lifestyle" OR "behavior" OR "Health behavior" OR "healthy lifestyle index") OR (Diet" OR "nutrition" OR "food" OR "dietary" OR "eating" OR "Food Habits" OR "nutrient" OR "vegetarian" OR "vegan" OR " dietary intake" OR "dietary patterns)) OR ("vegetables" OR "fruits" OR "legumes" OR "potatoes" OR "Tomato" OR "Radish" OR "Carrot" OR "Lettuce" OR "Spinach" OR "Onion" OR "fats" OR "oil" OR "vegetable fat" OR "sugar" "sugary" OR "milk" OR "carbohydrate" OR "fibre" OR "sugars" OR "total fat" OR "trans-fatty acids" OR "cholesterol" OR "Cereal" OR "grain" OR "granary" OR "wholegrain" OR "wholewheat" OR "roots" OR "tubers" OR "pulses" OR "beans" OR "lentils" OR "chickpeas" OR "soy" OR "soya" OR "nuts" OR "peanut" OR "groundnut" OR "seeds" OR "meat" OR " red meat" OR "beef" OR "pork" OR "lamb" OR "poultry" OR "chicken" OR "turkey" OR "duck" OR "fish" OR "eggs" OR "bread" OR "oils" OR "shellfish" OR "seafood" OR "sugar" OR "syrup" OR "dairy" OR "herbs" OR "spices" OR "chilli" OR "chillis" OR "pepper" OR "condiments" OR "Cabbage" OR "Brassica" OR "Cruciferous" OR "Soybean")) OR ("drink" OR "drinking" OR "fluid intake" OR "water" OR "sugar sweetened beverages" OR "tea" OR "coffee" OR "caffeine" OR "juice")) OR ("Cooking" OR "cooked" OR "grilled" OR "fried" OR "roast" OR "baked" OR "stewing" OR "stewed" OR "casserole" OR "broiled" OR "boiled" OR "microwaved" OR "reheating" OR "reheated" OR "heated" OR "poached" OR "steamed" OR "barbecue" OR "chargrill")) OR ("Macronutrients"[All Fields] OR "Micronutrients"[All Fields] OR "supplement"[All Fields] OR "vitamin"[All Fields] OR "retinol"[All Fields] OR "carotenoid"[All Fields] OR "tocopherol"[All Fields] OR "folate"[All Fields] OR "folic acid"[All Fields] OR "methionine"[All Fields] OR "riboflavin"[All Fields] OR "thiamine"[All Fields] OR "niacin"[All Fields] OR "pyridoxine"[All Fields] OR "cobalamin"[All Fields] OR "mineral"[All Fields] OR "sodium"[All Fields] OR "iron"[All Fields] OR "calcium"[All Fields] OR "selenium"[All Fields] OR "iodine"[All Fields] OR "magnesium"[All Fields] OR "potassium"[All Fields] OR "zinc"[All Fields] OR "copper"[All Fields] OR "phosphorus"[All Fields] OR "manganese"[All Fields] OR "chromium"[All Fields] OR "protein"[All Fields] OR "vitamin A"[All Fields] OR "carotenoids"[All Fields] OR "folate"[All Fields] OR "riboflavin"[All Fields] OR "vitamin B6"[All Fields] OR "cobalamin"[All Fields] OR "vitamin C"[All Fields] OR "vitamin D"[All Fields] OR "vitamin E"[All Fields] OR "iron"[All Fields] OR "calcium"[All Fields] OR "selenium"[All Fields] OR "isoflavones"[All Fields] OR "dichlorodiphenyltrichloroethane"[All Fields] OR "polychlorinated"[All Fields] OR "biphenyls"[All Fields] OR "hexachlorocyclohexane"[All Fields] OR "hexachlorobenzen"[All Fields] OR "hexachlorobenzene"[MeSH Terms] OR "hexachlorobenzene"[All Fields] OR "hexachlorobenzenes"[All Fields]"OR"[All

	Fields] OR "polyphenol s"[All Fields] OR "polyphenoles"[All Fields] OR "polyphenolic"[All Fields] OR "polyphenolics"[All Fields] OR "polyphenols"[MeSH Terms] OR "polyphenols"[All Fields] OR "polyphenol"[All Fields])) OR ("salted" OR "fibre" OR "polysaccharide" OR "starch" OR "starchy" OR "carbohydrate" OR "lipid" OR "linoleic acid" OR "sterols" OR "stanols" OR "saccharin" OR "aspartame" OR "acesulfame" OR "cyclamates" OR "maltose" OR "mannitol" OR "sorbitol" OR "sucrose" OR "xylitol" OR "cholesterol" OR "protein" OR "hydrogenated dietary oils" OR "hydrogenated lard" OR "hydrogenated oils")) OR ("mycotoxin" OR "aflatoxin" OR "pickled"OR "bottled" OR "bottling" OR "canned" OR "canning" OR "vacuum pack" OR "refrigerate" OR "refrigeration" OR "cured" OR "smoked" OR "preserved" OR "preservative" OR "nitrosamine" OR "hydrogenation" OR "fortified" OR "additive" OR "colouring" OR "coloring" OR "flavouring" OR "flavoring" OR "nitrates" OR "nitrites" OR "solvent" OR "solvents" OR "ferment" OR "processed" OR "antioxidant" OR "vinyl chloride" OR "packaging" OR "labelling" OR "phthalates"))
Science direct	breast cancer AND Diet AND and Middle east and North Africa
	"breast cancer" AND Nutrition AND MENA
Scopus	(TITLE-ABS-KEY (breast AND cancer) OR TITLE-ABS-KEY (breast AND neoplasms) OR TITLE-ABS-KEY (breast AND tumor) AND TITLE-ABS-KEY (lifestyle) OR TITLE-ABS-KEY (diet) OR TITLE-ABS-KEY (nutrition) OR TITLE-ABS-KEY (food) OR TITLE-ABS-KEY (nutrient) OR TITLE-ABS-KEY (vegetables) OR TITLE-ABS-KEY (fruits) OR TITLE-ABS-KEY (legumes) OR TITLE-ABS-KEY (potatoes) OR TITLE-ABS-KEY (drink) OR TITLE-ABS-KEY (macronutrients) OR TITLE-ABS-KEY (micronutrients) AND TITLE-ABS-KEY (meddle AND east) OR TITLE-ABS-KEY (north AND africa) OR TITLE-ABS-KEY (arabs) OR TITLE-ABS-KEY (eastern AND mediterranean) OR TITLE-ABS-KEY (algeria) OR TITLE-ABS-KEY (bahrain) OR TITLE-ABS-KEY (egypt) OR TITLE-ABS-KEY (iraq) OR TITLE-ABS-KEY (iran) OR TITLE-ABS-KEY (israel) OR TITLE-ABS-KEY (jordan) OR TITLE-ABS-KEY (kuwait) OR TITLE-ABS-KEY (lebanon) OR TITLE-ABS-KEY (libya) OR TITLE-ABS-KEY (morocco) OR TITLE-ABS-KEY (oman) OR TITLE-ABS-KEY (palestine) OR TITLE-ABS-KEY (qatar) OR TITLE-ABS-KEY (sudan) OR TITLE-ABS-KEY (syria) OR TITLE-ABS-KEY (saudi AND arabia) OR TITLE-ABS-KEY (turkey) OR TITLE-ABS-KEY (tunisia) OR TITLE-ABS-KEY (united AND arab AND emirates) OR TITLE-ABS-KEY (yemen) OR TITLE-ABS-KEY (mena))
Web of science	breast cancer (Title) and Diet (Title) or Nutrition (Title) or Dietary patterns (Title) and MENA (Title)

References

1. Soltani S, Benisi-Kohansal S, Azadbakht L, Esmaillzadeh A. Association between Adherence to "Dietary Approaches to Stop Hypertension" Eating Plan and Breast Cancer. Nutr Cancer. 2021;73(3):433–41.

2. Aghamohammadi V, Salari-Moghaddam A, Benisi-Kohansal S, Taghavi M, Azadbakht L, Esmaillzadeh A. Adherence to the MIND Diet and Risk of Breast Cancer: A Case-control Study. Clin Breast Cancer. 2021;21(3):e158–64.

3. Ahmadnia Z, Joukar F, Hasavari F, Roushan ZA, Khalili M. Dietary patterns and risk of breast cancer in women in Guilan Province, Iran. Asian Pac J Cancer Prev. 2016;17(4):2035–40.

4. Akbari A, Sohouli MH, Deliu Lozovanu O, Lotfi M, Nabavizadeh R, Saeidi R. Dietary insulin index and load with risk of breast cancer in a case-control study. Int J Clin Pract. 2021 Dec;75(12):e14883.

5. Al Qadire M, Alkhalaileh M, Hina H. Risk factors for breast cancer among Jordanian women: A case-control study. Iran J Public Health. 2018;47(1):49–56.

6. Alboghobeish Z, Hekmatdoost A, Jalali S, Ahmadi M, Rashidkhani B. Carbohydrate Intake, Glycemic Index, and Glycemic Load and the Risk of Breast Cancer among Iranian Women. Nutr Cancer. 2021;73(5):785–93.

7. Ali Ghalib HH, Ali DH, Molah Karim SA, Mohialdeen Gubari MI, Mohammed SA, Marif DH, et al. Risk factors assessment of breast cancer among Iraqi Kurdish women: Case-control study. J Fam Med Prim Care. 2019 Dec;8(12):3990–7.

8. Alim NE, Kiziltan G. Assessment of risk factors of obesity and diet on breast cancer in Ankara, Turkey. Pak J Med Sci. 2016;32(6):1537–42.

9. Allahyari P, Ahmadzadeh M, Vahid F, Gholamalizadeh M, Shafaei H, Shekari S, et al. The association of dietary antioxidant index (DAI) with breast cancer among Iranian women a case-control study. Int J Vitam Nutr Res 2022, 93, 483–489.

10. Alothaimeen A, Ezzat A, Mohamed G, Muammar T, Al-Madouj A. Dietary fat and breast cancer in Saudi Arabia: A case-control study. East Mediterr Health J. 2004;10(6):879–86.

11. Bidgoli SA, Azarshab H. Role of vitamin D deficiency and lack of sun exposure in the incidence of premenopausal breast cancer: A case control study in Sabzevar, Iran. Asian Pac J Cancer Prev. 2014;15(8):3391–6.

12. Azzeh FS, Hasanain DM, Qadhi AH, Ghafouri KJ, Azhar WF, Ghaith MM, et al. Consumption of Food Components of the Mediterranean Diet Decreases the Risk of Breast Cancer in the Makkah Region, Saudi Arabia: A Case-Control Study. Front Nutr. 2022;9:863029.

13. Bahadoran Z, Karimi Z, Houshiar-Rad A, Mirzayi HR, Rashidkhani B. Is dairy intake associated to breast cancer? A case control study of Iranian women. Nutr Cancer. 2013;65(8):1164–70.

14. Marzbani B, Nazari J, Najafi F, Marzbani B, Shahabadi S, Amini M, et al. Dietary Patterns, Nutrition, and Risk of Breast Cancer: A Case-Control Study in the West of Iran. Epidemiol Health. 2019;41:e2019003.

15. Dashti F, Soltani S, Benisi-Kohansal S, Azadbakht L, Esmaillzadeh A. Consumption of dairy products and odds of breast cancer: an Iranian case–control study. Breast Cancer. 2022;29(2):352–60.

16. Ebrahimpour-Koujan S, Benisi-Kohansal S, Azadbakht L, Esmaillzadeh A. The Association between Dietary Calcium Intake and Breast Cancer Risk among Iranian Women. Nutr Cancer. 2022;74(5):1652–9.

17. Ebrahimpour-Koujan S, Benisi-Kohansal S, Azadbakht L, Fallah M, Esmaillzadeh A. Adherence to HEI-2010 and odds of breast cancer according to the menopause status: Evidence from Middle Eastern Country. PloS One. 2024;19(3):e0300986.

18. Ceber E, Sogukpinar N, Mermer G, Aydemir G. Nutrition, lifestyle, and breast cancer risk among Turkish women. Nutr Cancer. 2005;53(2):152–9.

19. Fararouei M, Iqbal A, Rezaian S, Gheibi Z, Dianatinasab A, Shakarami S, et al. Dietary Habits and Physical Activity are Associated With the Risk of Breast Cancer Among Young Iranian Women: A Case-control Study on 1010 Premenopausal Women. Clin Breast Cancer. 2019;19(1):e127–34.

20. Vahid F, Shivappa N, Hatami M, Sadeghi M, Ameri F, Naeini YJ, et al. Association between dietary inflammatory index (DII) and risk of breast cancer: A case-control study. Asian Pac J Cancer Prev. 2018;19(5):1215–21.

21. Vahid F, Rahmani W, Khodabakhshi A, Davoodi SH. Associated Between Dietary Antioxidant Index (dai) and Odds of Breast Cancer and Correlation Between Dai with Pathobiological Markers: Hospital-Based Incidence Case-Control Study. J Am Nutr Assoc. 2023 Jun;42(4):386–92.

22. Djafari F, Ghorbaninejad P, Firouzabadi FD, Sheikhhossein F, Shahinfar H, Safabakhsh M, et al. Adherence to Mediterranean dietary quality index and risk of breast cancer in adult women: a case-control study. BMC Womens Health. 2023 Mar 14;23(1):107.

23. Fereidani SS, Eini-Zinab H, Heidari Z, Jalali S, Sedaghat F, Rashidkhani B. Nutrient patterns and risk of breast cancer among Iranian Women: A case- control study. Asian Pac J Cancer Prev. 2018;19(9):2619–24.

24. Foroozani E, Akbari A, Amanat S, Rashidi N, Bastam D, Ataee S, et al. Adherence to a western dietary pattern and risk of invasive ductal and lobular breast carcinomas: a case–control study. Sci Rep. 2022 Apr 7;12(1):5859.

25. Ghanbari M, Shahinfar H, Imani H, Safabakhsh M, Shab-Bidar S. Association of Empirically Derived Food-Based Inflammatory Potential of the Diet and Breast Cancer: A Hospital-Based Case-Control Study. Clin Breast Cancer. 2022;22(4):e567–75.

26. Gholamalizadeh M, Shahdoosti H, Bahadori E, BourBour F, Akbari ME, Rastgoo S, et al. Association of different types of dietary fatty acids with breast cancer, a case-control study. Nutr Food Sci. 2022;52(3):561–8.

27. Gholamalizadeh M, Afsharfar M, Fathi S, Tajadod S, Mohseni GK, Shekari S, et al. Relationship between breast cancer and dietary inflammatory index; a case-control study. Clin Nutr ESPEN. 2022 Oct;51:353–8.

28. Hammad SS, Mahmoud R, Marie L, Abdelrahim D, Tayyem RF. Association between grain and legume intakes and breast cancer risk among women. Ann Cancer Res Ther. 2020;28(2):81–7.

29. Hammad SS, Mahmoud R, Shivappa N, Hebert JR, Marie L, Tayyem RF. Dietary inflammatory index and odds of breast cancer: A case–control study. Food Sci Nutr. 2021;9(9):5211–9.

30. Hayati Z, Montazeri V, Shivappa N, Hebert JR, Pirouzpanah S. The association between the inflammatory potential of diet and the risk of histopathological and molecular subtypes of breast cancer in northwestern Iran: Results from the Breast Cancer Risk and Lifestyle study. Cancer. 2022 Jun 15;128(12):2298–312.

31. Heidari Z, Jalali S, Sedaghat F, Ehteshami M, Rashidkhani B. Dietary patterns and breast cancer risk among Iranian women: A case-control study. Eur J Obstet Gynecol Reprod Biol. 2018;230:73–8.

32. Heidari Z, Mohammadi E, Aghamohammadi V, Jalali S, Rezazadeh A, Sedaghat F, et al. Dietary Approaches to Stop Hypertension (DASH) diets and breast cancer among women: A case control study. BMC Cancer [Internet]. 2020;20(1). Available from: https://www.scopus.com/inward/record.uri?eid=2-s2.0-85088852632&doi=10.1186%2fs12885-020-07209-1&partnerID=40&md5=5a1bcb780579e2486b4d130436857133

33. Hosseini F, Imani H, Sheikhhossein F, Majdi M, Ghanbari M, Shab-Bidar S. Dietary Carbohydrate Quality and Quantity and Risk of Breast Cancer among Iranian Women. Nutr Cancer. 2022;74(3):916–26.

34. Hosseinzadeh M, Ziaei JE, Mahdavi N, Aghajari P, Vahidi M, Fateh A, et al. Risk factors for breast cancer in iranian women: A hospital-based case-control study in Tabriz, Iran. J Breast Cancer. 2014;17(3):236–43.

35. Jalali S, Shivappa N, Hébert JR, Heidari Z, Hekmatdoost A, Rashidkhani B. Dietary Inflammatory Index and Odds of Breast Cancer in a Case-Control Study from Iran. Nutr Cancer. 2018;70(7):1034–42.

36. Jalali S, Heidari Z, de Courten B, Rashidkhani B. Dietary Total Antioxidant Capacity and Odds of Breast Cancer: A Case-Control Study. Nutr Cancer. 2023;75(1):302–9.

37. Jamshidinaeini Y, Akbari ME, Abdollahi M, Ajami M, Davoodi SH. Vitamin D Status and Risk of Breast Cancer in Iranian Women: A Case–Control Study. J Am Coll Nutr. 2016;35(7):639–46.

38. Karimi Z, Jessri M, Houshiar-Rad A, Mirzaei HR, Rashidkhani B. Dietary patterns and breast cancer risk among women. Public Health Nutr. 2014;17(5):1098–106.

39. Karimi Z, Bahadoran Z, Abedini S, Houshyar-Rad A, Rashidkhani B. Dietary total antioxidant capacity and the risk of breast cancer: A case–control study. East Mediterr Health J. 2015;21(8):564–71.

40. Laamiri FZ, Bouayad A, Otmani A, Ahid S, Mrabet M, Barkat A. Dietery factor obesity microenvironnement and breast cancer. Gland Surg. 2014 Aug;3(3):165–73.

41. Zahedi A, Lotfi MH, Yavari P, Mahboubi M, Rastegar Mehr B, Jafaee Sough A, et al. Dietary and individual habits in women with breast cancer in Yazd Province. Int J Pharm Technol. 2015;7(1):8196–209.

42. Maliou D, Belmadi D, Saadi W, Mahfouf H, Benzidane N, Bitam A. Effect of dairy products intake on breast cancer risk: A case-control study in Algeria. Nutr Clin Metab. 2018;32(3):187–94.

43. Mobarakeh ZS, Mirzaei K, Hatmi N, Ebrahimi M, Dabiran S, Sotoudeh G. Dietary habits contributing to breast cancer risk among Iranian women. Asian Pac J Cancer Prev. 2014;15(21):9543–7.

44. Mohammadzadeh M, Bahrami A, Abdi F, Ghafouri-Taleghani F, Paydareh A, Jalali S, et al. Dietary Diabetes Risk Reduction Score (DDRRS) and Breast Cancer Risk: A Case-Control Study in Iran. Nutr Cancer. 2024;76(1):106–13.

45. Ebrahimi Mousavi S, Bagheri A, Benisi-Kohansal S, Azadbakht L, Esmaillzadeh A. Consumption of "Diabetes Risk Reduction Diet" and Odds of Breast Cancer Among Women in a Middle Eastern Country. Front Nutr. 2022;9:744500.

46. Payandeh N, Shahinfar H, Amini MR, Jafari A, Safabakhsh M, Imani H, et al. The Lack of Association between Plant-Based Dietary Pattern and Breast Cancer: a Hospital-Based Case-Control Study. Clin Nutr Res. 2021 Apr;10(2):115–26.

47. Rigi S, Mousavi SM, Benisi-Kohansal S, Azadbakht L, Esmaillzadeh A. The association between plant-based dietary patterns and risk of breast cancer: a case–control study. Sci Rep. 2021 Feb 9;11(1):3391.

48. Rigi S, Salari-Moghaddam A, Benisi-Kohansal S, Azadbakht L, Esmaillzadeh A. Dietary glycaemic index and glycaemic load in relation to risk of breast cancer. Public Health Nutr. 2022;25(6):1658–66.

49. Sadeghi O, Eshaghian N, Benisi-Kohansal S, Azadbakht L, Esmaillzadeh A. A case–control study on the association between adherence to a Mediterranean-style diet and breast cancer. Front Nutr. 2023 Jul 18;10:1140014.

50. Safabakhsh M, Imani H, Shab-Bidar S. Higher dietary total antioxidant capacity is not associated with risk of breast cancer in Iranian women. Breast Cancer. 2020;27(4):652–61.

51. Safabakhsh M, Shab-Bidar S, Imani H. Higher Fruits and Vegetables Consumption Is not Associated with Risk of Breast Cancer in Iranian Women. Nutr Cancer. 2022;74(5):1680–91.

52. Sasanfar B, Toorang F, Esmaillzadeh A, Zendehdel K. Adherence to the low carbohydrate diet and the risk of breast Cancer in Iran. Nutr J [Internet]. 2019;18(1). Available from: https://www.scopus.com/inward/record.uri?eid=2-s2.0-85076420336&doi=10.1186%2fs12937-019-0511-x&partnerID=40&md5=c1f1d444552cd00f93fe96597b20d3f2

53. Sasanfar B, Toorang F, Maleki F, Esmaillzadeh A, Zendehdel K. Association between dietary total antioxidant capacity and breast cancer: A case-control study in a Middle Eastern country. Public Health Nutr. 2021;24(5):965–72.

54. Sasanfar B, Toorang F, Booyani Z, Vassalami F, Mohebbi E, Azadbakht L, et al. Adherence to plant-based dietary pattern and risk of breast cancer among Iranian women. Eur J Clin Nutr. 2021;75(11):1578–87.

55. Sasanfar B, Toorang F, Mohebbi E, Zendehdel K, Azadbakht L. Dietary carbohydrate quality and risk of breast cancer among women. Nutr J. 2021 Nov 26;20(1):93.

56. Shafie F, Tajadod S, Aslany Z, Allahyari P, Vahdat M, Shekari S, et al. Breast cancer and dietary fat quality indices in Iranian women: A case-control study. Front Oncol. 2022;12:993397.

57. Sharif Y, Sadeghi O, Benisi-Kohansal S, Azadbakht L, Esmaillzadeh A. Legume and Nuts Consumption in Relation to Odds of Breast Cancer: A Case-Control Study. Nutr Cancer. 2021;73(5):750–9.

58. Sheikhhossein F, Imani H, Amini MR, Hosseini F, Shab-Bidar S. The association between adherence to MIND diet and risk of breast cancer: A case–control study. Int J Clin Pract [Internet]. 2021;75(11). Available from: https://www.scopus.com/inward/record.uri?eid=2-s2.0-85115056601&doi=10.1111%2fijcp.14780&partnerID=40&md5=f99b13753c92285281c08fa3f1438919

59. Sheikhhossein F, Shab-Bidar S, Amini MR, Hosseini F, Imani H. Dietary Insulin Index and Insulin Load in Relation to Breast Cancer: Findings from a Case–Control Study. Clin Breast Cancer. 2021;21(6):e665–74.

60. Sohouli MH, Hadizadeh M, Mardali F, Sanati V, da Silva Magalhães EI, Zarrati M. Association between novel dietary and lifestyle inflammation indices with risk of breast cancer (BrCa): a case–control study. Nutr J [Internet]. 2022;21(1). Available from: https://www.scopus.com/inward/record.uri?eid=2-s2.0-85125595656&doi=10.1186%2fs12937-022-00766-0&partnerID=40&md5=b47986f5f32beec9efef2f6eedbecf12

61. Tajaddini A, Pourzand A, Sanaat Z, Pirouzpanah S. Dietary resistant starch contained foods and breast cancer risk: A case-control study in Northwest of Iran. Asian Pac J Cancer Prev. 2015;16(10):4185–92.

62. Tayyem RF, Mahmoud RI, Shareef MH, Marei LS. Nutrient intake patterns and breast cancer risk among Jordanian women: a case-control study. Epidemiol Health. 2019;41:e2019010.

63. Tiznobeyk Z, Sheikhi Mobarakeh Z, Qorbani M, Koohdani F, Sotoudeh G, Khajehnasiri F, et al. Dietary patterns and benign breast diseases: A case-control study. Br J Nutr. 2016;116(2):353–9.

64. Toklu H, Nogay NH. Effects of dietary habits and sedentary lifestyle on breast cancer among women attending the oncology day treatment center at a state university in Turkey. Niger J Clin Pract. 2018;21(12):1576–84.

65. Toorang F, Sasanfar B, Esmaillzadeh A, Zendehdel K. Adherence to the DASH Diet and Risk of Breast Cancer. Clin Breast Cancer. 2022;22(3):244–51.
